# Functional characterization of 58-kilodalton inhibitor of protein kinase in protecting against diabetic retinopathy via the endoplasmic reticulum stress pathway

**Published:** 2011-01-10

**Authors:** Hong Yang, Rong Liu, Zheng Cui, Zhi-qi Chen, Shu Yan, Han Pei, Bin Li

**Affiliations:** Department of Ophthalmology, Tongji Hospital, Tongji Medical College, Huazhong University of Science and Technology, Wuhan, Hubei Province, People’s Republic of China

## Abstract

**Objective:**

58-kilodalton inhibitor of protein kinase (P58^IPK^) plays an important role in preventing endoplasmic reticulum (ER) stress. It is an interferon-induced kinase that targets the eukaryotic translation initiation factor eukaryotic initiation factor 2 alpha. The aim of this study was to determine the roles of P58^IPK^ in protecting against diabetic retinopathy (DR) by inhibiting ER stress-signaling mediators.

**Methods:**

A rat diabetic model was established by intraperitoneal injection of streptozotocin. Overexpression of P58^IPK^ was achieved by intravitreal injection of purified recombinant adeno-associated virus vector (rAAV2)-P58^IPK^ or transfection into rat retinal capillary endothelial cells. Retinal vascular permeability was determined by assessing the Evans Blue retinal leakage. To downregulate the P58^IPK^ level in cultured rat retinal capillary endothelial cells, pGIPZ-P58^IPK^ RNA interference (P58^IPK^RNAi) was introduced in these cells. Real time reverse transcription (RT)–PCR and western blot analyses were performed to evaluate the mRNA and protein levels of Core/emopamil binding protein (C/EBP) homologous protein (CHOP), vascular endothelial growth factor (VEGF), and tumor necrosis factor-α (TNF-α).

**Results:**

Retinal blood vessel leakage was significantly decreased in rAAV2-P58^IPK^-transfected diabetic rats compared with the control diabetic rats. Both mRNA and protein levels of CHOP, TNF-α, and VEGF in the retina of diabetic rats were remarkably reduced in P58^IPK^-transfected rats. In vitro study further demonstrated that overexpression of P58^IPK^ downregulated the expression of CHOP, TNF-α, and VEGF under high glucose conditions, whereas introduction of P58^IPK^RNAi enhanced the expression of CHOP, TNF-α, and VEGF.

**Conclusions:**

These results revealed the protecting role of P58^IPK^ against ER stress-mediated DR in diabetic rats, suggesting that P58^IPK^ may act as a DR-resistant gene during diabetes.

## Introduction

Diabetic retinopathy (DR) is a common complication of diabetes. Owing to the increased number of people with diabetes worldwide, DR has become the most frequent cause of postnatal blindness in people of working age in industrialized countries [1]. Although the precise mechanism of DR is poorly understood, accumulating evidence has revealed that endoplasmic reticulum (ER) stress is involved in the death of both retinal neurons and vascular cells in diabetic eyes, play an important role in the DR [2].

Various factors have been implicated in ER stress-mediated DR. Our previous study demonstrated that the expression of Core/emopamil binding protein (C/EBP) homologous protein (CHOP), an ER stress-induced transcription factor, was increased during the early stage of DR [3]. In addition, the expression of tumor necrosis factor (TNF)-α was significantly upregulated in the retina of a genetic mouse model of type I diabetes [4]. P58^IPK^, a member of the heat shock protein 40 (Hsp40) family, phosphorylates eukaryotic initiation factor 2α (elF-2α) and sequesters tis sequesters its guanosine diphosphate (GDP)/guanosine triphosphate (GTP) exchange factor [5]. It has been suggested that P58^IPK^ plays an important role in preventing ER stress [6,7]. Overexpression of P58^IPK^ in human retinal capiliry endothail cells (HRCECs) downregulated the expression of vascular endothelial growth factor (VEGF) and CHOP and effectively suppressed ER stress [8]. Microarray analysis with blood samples from diabetic patients demonstrated enhanced P58^IPK^ gene expression patients without proliferative diabetic retinopathy compared with patients with proliferative diabetic retinopathy onset [9], suggesting that P58^IPK^ may exert a potential role in DR resistance. In the present study we investigated the roles of P58^IPK^ in protecting against ER stress-mediated DR and the mechanisms involved.

## Methods

### Overexpression of P58^IPK^ in rat retina

We obtained eighty two-month-old male Sprague Dawley rats, weighing between 150 and 200 g, from the animal center of Huazhong University of Science and Technology, and maintained in an optimal environment for 2 weeks before experimentation. Rats were randomly divided into four groups, A, B, C, and D, with 20 rats in each group. Each rat in group A and B received an intravitreal injection of purified recombinant adeno-associated virus vector- green fluorescent protein (rAAV2-GFP) in either the left or right eye (determined randomly). Rats in group C and D were injected with rAAV2-P58^IPK^ [8]. The diabetic animal model was established 1 month after rAAV2 transfection to retina of rats in groups A and C. Following fasting for 12 h, the rats were intraperitoneally injected with a single dose of streptozotocin (STZ; 65 mg/kg in 0.01 mol citrate buffer with a pH of 4.5). Nondiabetic control mice received citrate buffer only. Collected blood from tail vein, glucose testing Used the glucose test strips (Roche diagnostics-China company, Shanghai, China) 3 days after STZ injection, and diabetes was confirmed by a fasting plasma glucose value of 16.7 mmol/l or higher using a Touch™ Glucometer (Boehringer Mannheim Diagnostics, Indianapolis, IN). Retinal integrity was assessed 2 months later. In brief, a total of four experimental groups were set in this study, GFP/diabetes group, GFP/no-dia group, P58^IPK^/dia group, and P58^IPK^/no-dia group.

### Assessment of retinal blood vessel leakage

Retinal vascular permeability was quantified by measuring the Evans Blue (EB) retinal leakage [9]. Briefly, 45 mg/kg of 3% EB (Sigma, Carlsbad, CA) was injected via the tail vein. Two hours later, under the anesthesia with the injection of chloral hydrate 10% (3ml/kg) in abdominal cavity, the chest of the rat was opened and an infusion tube connected with a 14G blunt needle was inserted into the left ventricle. The right atrial appendage was opened, and the heart was infused with the fluid containing 1% paraformaldehyde in 0.05 M citrate buffer (pH 3.5; 250 ml/kg bodyweight). Infusion height was 160 cm. The infusion was maintained for 2 min to remove any remnant dye in the blood vessels. The eyes of the rat were then removed. Dissected retinas were dried under a vacuum at 45 °C for 5 h. The weight of dried retina was then recorded. The difference in retinal weight between diabetic rats injected with rAAV2-P58^IPK^ and controls was compared. Next, 120 μl of formamide was added to the samples, and the mixture was placed in a 70 °C water bath for 18 h. The sample was then centrifuged at 7,280× g for 30 min to separate the dye from the protein. The absorbance of the supernatant was at wavelengths of 620 nm and 740 nm using a Beckman DU-640 spectrophotometer (Beckman Coulter, Fullerton, CA). The net absorbance was calculated by subtracting the absorbance at 740 nm from that at 620 nm. The concentration of dye was calculated from a standard curve (determined for each measurement) of EB in formamide. Samples were analyzed in triplicate, and the mean value was calculated. The weight of the dry retina (mg) was used to standardize dye content; values are presented in ng/mg. The formula was as follows: EB content in the retina (ng/mg)=[EB concentration in formamide (ng/μl)×20 (μl)]/weight of the dry retina (mg).

### Preparation and cultivation of rat retinal capillary endothelial cells

Rat eyes were cut circumferentially 1.5 mm posterior to the limbus, and the retinas were harvested and homogenized by two gentle up-and-down strokes in a 15-ml homogenizer (Dounce; Bellco Glass Co., Vineland, NJ). The homogenate was filtered, and the remaining retentate was digested in 0.066% collagenase (Invitrogen Inc., Carlsbad, CA) at 37 °C for 45 min. The homogenate was centrifuged (1,000× g for 10 min), and the pellet was resuspended in human serum-free endothelial basal growth medium (Invitrogen–Gibco, Grand Island, NY), supplemented with 20% fetal bovine serum and 50 U/ml endothelial cell growth factor (Sigma-Aldrich, St. Louis, MO). Cells were cultured in fibronectin-coated dishes and incubated at 37 °C in a humidified atmosphere containing 5% CO_2_. To investigate the expression of P58^IPK^, cultured rat retinal capillary endothelial cells (RRCECs) were transfected with pGIPZ-P58^IPK^ RNAi (group A), pGIPZ-GFP (control group, group B), irrelevant small interfering RNA (siRNA; group C), and rAAV2-P58^IPK^ (group D). Real-time PCR and western blot were used to evaluate the expression of P58^IPK^ in RRCECs.

### Experimental groups

Cells were plated in 24-well culture plates and incubated overnight to 70%–80% confluence. Cells were then washed twice with human endothelial serum-free basal growth medium. Cells were randomly divided into four groups: 1, 2, 3, and 4. Then, 1×10^9^ μg/ml pGIPZ-P58^IPK^ RNAi was added to group 1, and cells were maintained in culture medium supplemented with 30 mM/ml glucose (RNAi/high-glu); 1×10^9^ μg/ml rAAV2-P58^IPK^ was added to group 2, and cells were maintained in culture medium supplemented with 30 mM/ml glucose (P58^IPK^/high-glu); 1×10^9^ μg/ml pGIPZ-P58^IPK^ RNAi was added to group 3, and cells were cultured with normal human vascular endothelial cell culture media (RNAi/normal-glu); 1×10^9^ μg/ml rAAV2-P58^IPK^ was added to group 4, and cells were cultured with normal human vascular endothelial cell culture media (P58^IPK^/normal-glu).

### Real-time PCR

Total RNA was isolated using RNeasy mini Kit (Qiagen, Hong Kong, China) from 30 mg of homogenized tissues with a kit (Qiagen, Hong Kong, China) according to the manufacturer’s protocol. cDNA was then transcribed using a Super Script II RT kit (Invitrogen). Real-time real-time PCR -PCR was performed with a *Taq* polymerase kit (Hotstar; Qiagen) using SYBR Green technology (Applied Biosystems Inc., Foster City, CA). β-actin was used as an internal control for the PCRs. Primers for PCRs are presented in Table 1. The PCR reaction for P58^IPK^ was performed in a final reaction volume of 50 μl in the following conditions: a preheating cycle at 94 °C for 3 min, followed by 30 cycles at 94 °C for 1 min, 56 °C for 30 s, and 72 °C for 45 s, and finally elongated at 72 °C for 8 min. The PCR conditions for VEGF, TNF-α, and CHOP were similar, except for the annealing temperature; VEGF was 58 °C, while TNF-α and CHOP were 56 °C. A melting curve analysis was performed by monitoring FAM/SYBR fluorescence.

**Table 1:** Real-time PCR primers used for assessing gene expression in rat retina tissue.

**Target gene**	**Primers**	**Annealing temperature (°C)**
*P58^IPK^*	F: ATTAAAGCATACCGAAAGTTAGCAC	56
	R: AGAGGGTCTTCTCCGTCATCAAA	
*TNF-α*	F: GGCAGCCTTGTCCCTTGAAGAG	56
	R: GTAGCCCACGTCGTAGCAAACC	
*CHOP*	F: ACCTTCACTACTCTTGACCCTG	56
	R: TCATTCTCCTGCTCCTTCT	
*VEGF*	F: TCTTCAAGCCGTCCTGTGTG	58
	R: ACAGTGAACGCTCCAGGATTTA	
*ACTB*	F: CTGGGTATGGAATCCTGTGG	56
	R: TCATCGTACTCCTGCTTGCTG	

### Western blotting analysis

Total protein extracted from the rat retinas or RRCECs was quantified using bicinchoninic acid (Sigma Aldrich). The protein was mixed with the loading buffer, denatured for 6 min at 60 °C, cooled, centrifuged 12,000× g for 5 min, and then separated by sodium-dodecyl sulfate PAGE (PAGE). Monoclonal anti-TNF-α (Santa Cruz Biotechnology Inc., Santa Cruz, CA), polyclonal anti-CHOP (Santa Cruz Biotechnology, Inc.), and anti-VEGF (Santa Cruz Biotechnology, Inc.) antibodies were used to probe the proteins. Some of the same polyvinylidene fluoride membranes were reused to detect β-actin (internal control) by incubating them with the mouse antihuman actin antibody (Gene Co., Hong Kong, China). The bands observed on the films were analyzed by automatic image analysis Odyssey scanner (Li-Cor, Lincoln, NE), and the integrated optical density of each protein band was normalized to the integrated optical density value of the corresponding β-actin band from the same sample.

### Statistical analyses

Normally distributed data were compared by independent two-sample *t* test or one-way ANOVA where appropriate. When a significant difference was detected between groups, multiple comparisons of means were performed using the Bonferroni procedure, with the type I error rate at a maximum of 0.017 (0.05/3) adjustment. Statistical analyses were performed using SPSS 15.0 statistical software (SPSS Inc., Chicago, IL). Data were presented as the mean±standard deviation (SD). A p value <0.05 was considered to be significant.

## Results

### Overexpression of P58^IPK^ reduced retinal blood vessel leakage in diabetic rats

**Figure 1:**
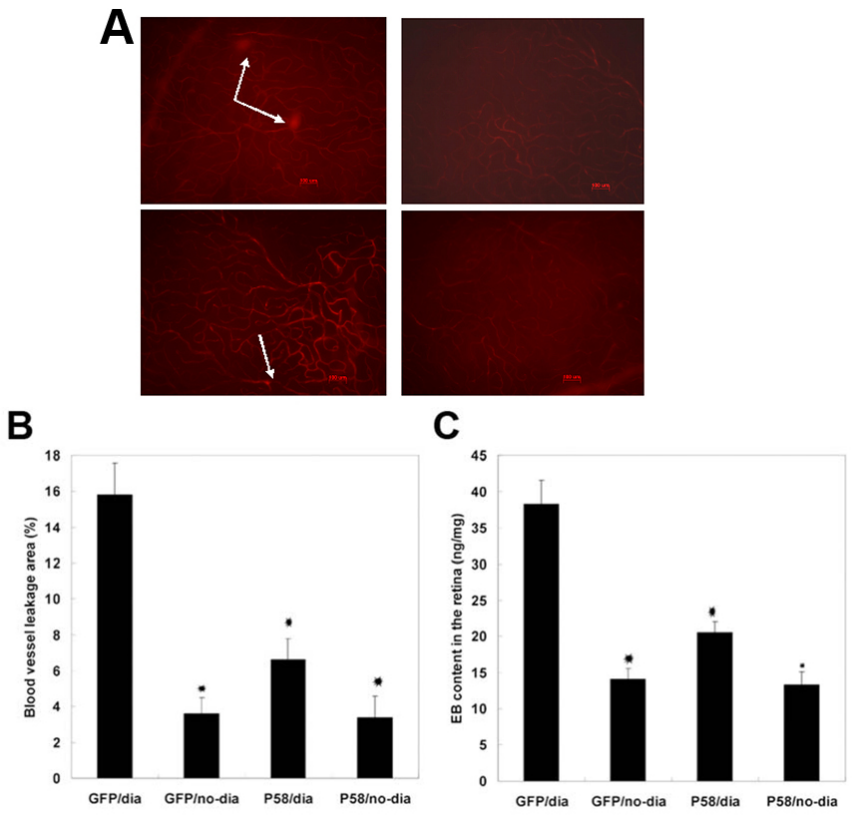
Measurement of blood–retinal barrier breakdown by Evans blue. **A**: Representative microphotographs from groups GFP/dia, GFP/no-dia, P58/dia, and P58/no-dia. Scale bar equal to 100 μm for **A**-**D**. **B**: The areas of blood vessel leakage were quantified and presented as mean±standard deviation; 15.8±1.8% in the GFP/dia group (n=6); 3.6±0.9% in the GFP/no-dia group (n=6); 6.6±1.2% in the P58^IPK^/dia group (n=6); and 3.4±1.2% in the P58/no-dia group (n=6; p<0.05, GFP/dia group versus P58/dia group); * There was a significant difference that GFP/dia Group compared with GFP/no-dia group, P58/dia group and P58/no-dia group (p<0.05; n=6).  **C**: EB contents in the rat retina were evaluated as described in Methods. EB contents in the retina obtained from rAAV2-P58^IPK^-infected rats were lower compared with the GFP/dia group: 38.3±3.3 ng/mg in the GFP/dia group; 14.1±1.5 ng/mg in the GFP/no-dia  group; 20.5±1.5 ng/mg in the P58/dia group; and 13.3±1.8 ng/mg in the P58^IPK^/non-dia group (p<0.05, GFP/dia group versus P58^IPK^/dia group),* There was a significant difference that GFP/dia Group compared with GFP/no-dia group, P58/dia group and P58/no-dia group (p<0.05; n=6).

A rat diabetes model was induced by intraperitoneal injection of STZ. Rats with a fast blood glucose concentration in excess of 16.7 mmol/l were considered to be diabetic. Vascular permeability was measured by retinal EB dye accumulation. As shown in Figure 1A, retinal blood vessel leakage was significantly decreased in diabetic rats injected with rAAV2-P58^IPK^ compared with the diabetic rats without P58^IPK^ (rAAV2-GFP). Quantified data revealed that the area of blood vessel leakage was markedly reduced: 15.8±1.8% in the GFP/dia group (n=6); 3.6±0.9% in the GFP/no-dia group (n=6); 6.6±1.2% in the P58IPK/dia group (n=6); and 3.4±1.2% in the P58/no-dia group (n=6; p<0.05, GFP/dia group versus P58/dia group; Figure 1B). Similarly, EB contents in the retina obtained from rAAV2-P58^IPK^-infected rats were lower compared with the GFP/dia group: 38.3±3.3 ng/mg in the GFP/dia group; 14.1±1.5 ng/mg in the GFP/no-dia group; 20.5±1.5 ng/mg in the P58/dia group; and 13.3±1.8 ng/mg in the P58^IPK^/no-dia group (p<0.05, GFP/dia group versus P58^IPK^/dia group, Figure 1C). These data demonstrate that P58^IPK^ overexpression inhibited the retinal blood vessel leakage in diabetic rats (Figure 1).

### Overexpression of P58^IPK^ suppressed the expression of CHOP, TNF-α, and VEGF in the retina of diabetic rats

**Figure 2:**
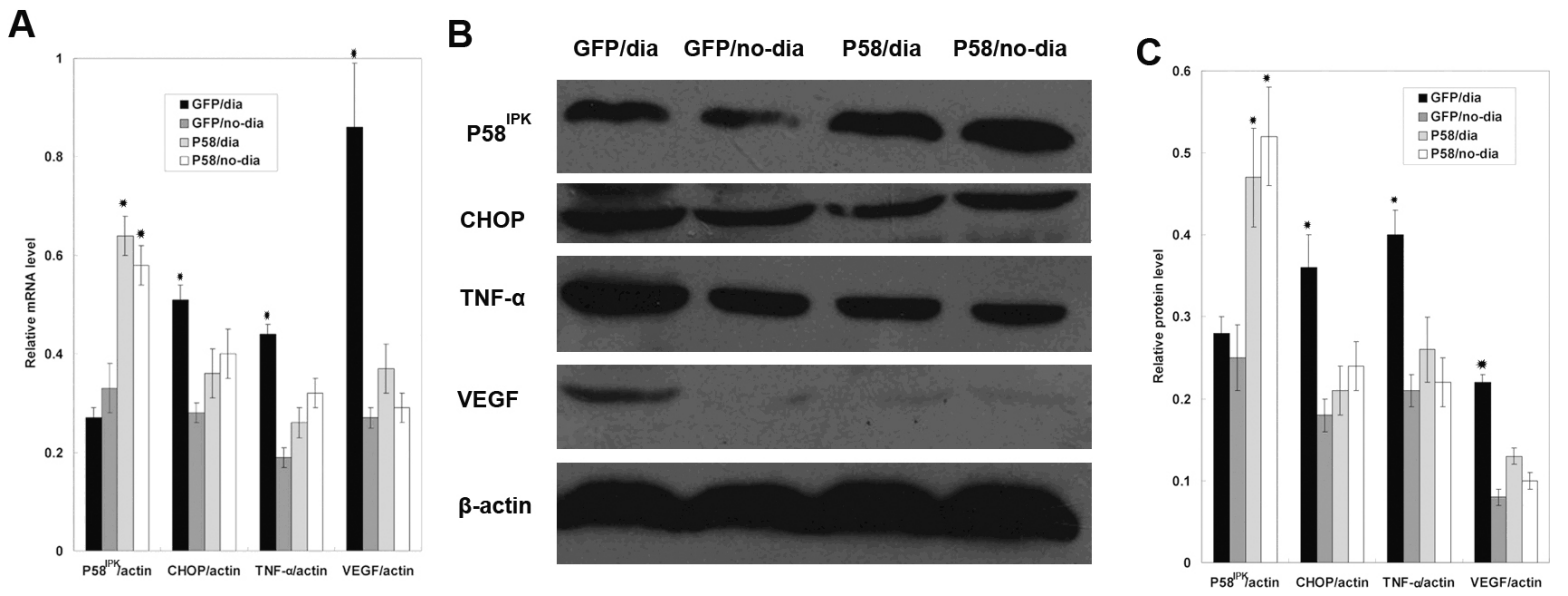
Overexpression of P58^IPK^ suppressed the expression of Core/emopamil binding protein homologous protein (CHOP), tumor necrosis factor (TNF)-α and vascular endothelial growth factor (VEGF) in the retina of diabetic rats. **A**: mRNA levels of P58^IPK^, CHOP, TNF-α, and VEGF were determined by real-time PCR, in GFP/dia group, GFP/no-dia group, P58^IPK^/dia group, and P58^IPK^/no-dia group, The expression level of P58^IPK^ in each groups were 0.27±0.02, 0.33±0.05, 0.64±0.04, and 0.58±0.04 (p<0.05, n=6), The expression level of CHOP in each groups were 0.51±0.03, 0.28±0.02, 0.36±0.05, and 0.40±0.05; respectively, (p<0.05, n=6), The expression level of TNF-α in each groups were 0.44±0.02, 0.19±0.02, 0.26±0.03, and 0.32±0.03 respectively, (p<0.05, n=6), The expression level of VEGF in each groups were 0.86±0.13, 0.27±0.02, 0.37±0.05, and 0.29±0.03 respectively, (p<0.05, n=6),  **B**: A representative western blot result is shown in **B**. **C**: The amount of protein expression was quantified relative to the level of β-actin. Results are representative of six experiments. Results are expressed as mean±standard deviation; The protein level of P58^IPK^ in each groups were 0.28± 0.02, 0.25±0.04, 0.47±0.064, and 0.52±0.06 (p<0.05, n=6), The protein level of CHOP in each groups were 0.36 ±0.04, 0.18±0.02, 0.21±0.03, and 0.24±0.03;respectively, (p<0.05, n=6), The protein level of TNF-α in each groups were 0.40±0.03, 0.21±0.02, 0.26±0.04, and 0.22±0.03 respectively, (p<0.05, n=6), The protein level of VEGF in each groups were 0.22±0.01, 0.08±0.01, 0.13±0.01, and 0.10±0.01 respectively, (p<0.05, n=6).

Our previous study showed that the level of ER stress-induced transcription factor CHOP was remarkably increased during the early stage of DR [3]. In addition, the expression of TNF-α was found unregulated in the retina of a genetic mouse model of type 1 diabetes [4]. Based on these observations, we investigated the effects of P58^IPK^ on mRNA and protein levels of CHOP, TNF-α, and VEGF, using real-time PCR and western blot analyses. As shown in Figure 2, P58^IPK^ overexpression was observed in the retina of diabetic rats. Interestingly, overexpression of P58^IPK^ remarkably suppressed the mRNA and protein levels of CHOP, TNF-α, and VEGF compared to control groups (Figure 2A-C).

### Knockdown of endogenous P58^IPK^ enhanced the expression of CHOP, TNF-α, and VEGF in RRCECs

**Figure 3:**
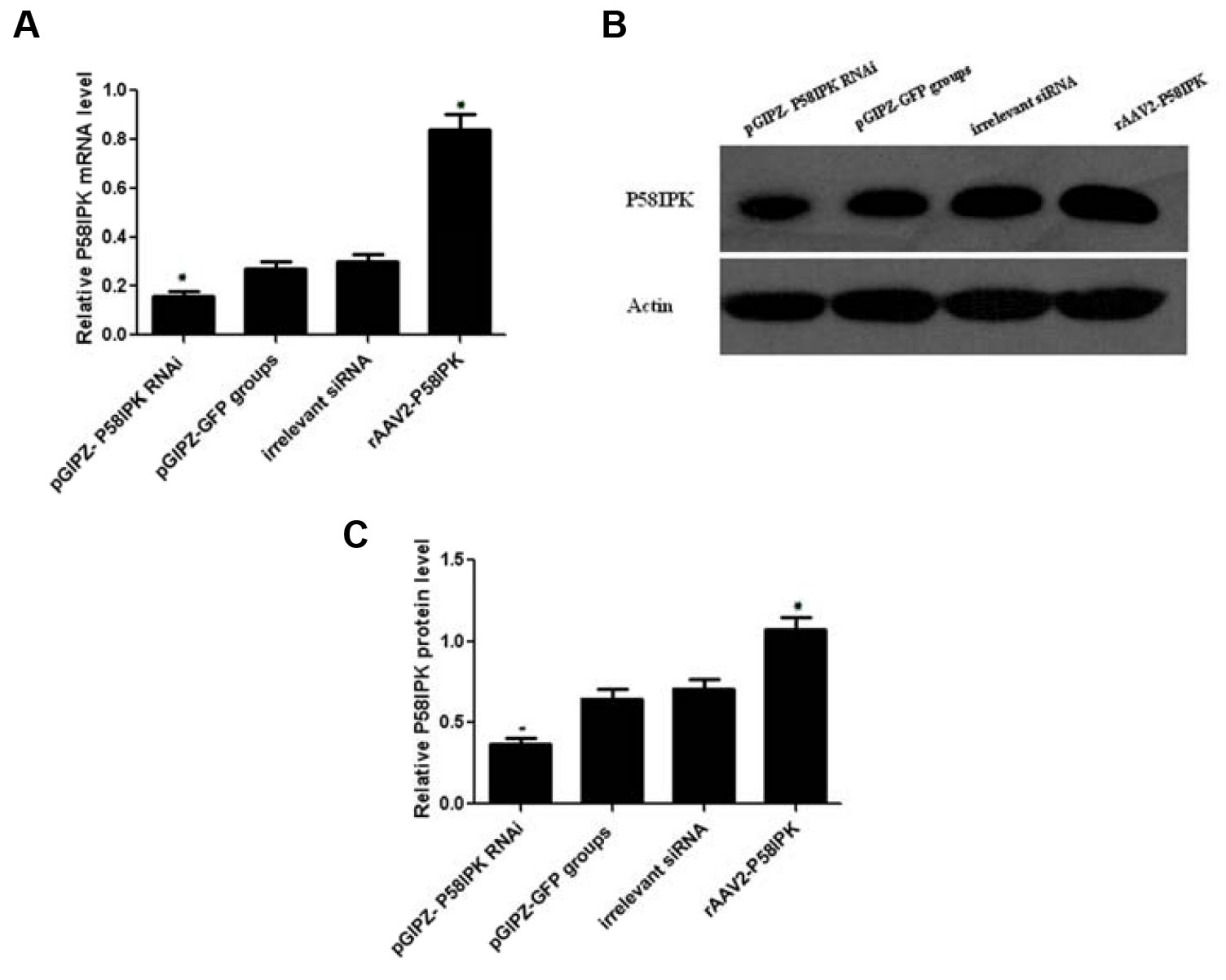
The expression of P58^IPK^ in cultured RRCECs in group A (pGIPZ-P58^IPK^ RNAi), group B (control group), group C (irrelevant siRNA), and group D (rAAV2-P58^IPK^). **A**: mRNA levels of P58^IPK^ were determined by real-time PCR analysis. The expression level of P58^IPK^ in either groups were 0.16±0.02, 0.27±0.03, 0.30±0.03, 0.84±0.06 respectively, There was a significant difference that Group B compared with group A and group D, (p<0.05; n=6). There was not a significant difference that Group B compared with group C, (p>0.05; n=6).  **B**: A representative western blot result. **C**: The amount of protein expression was quantified relative to the level of β-actin. Results are representative of six experiments. Results are expressed as mean±standard deviation; The protein level of P58^IPK^ in either groups were 0.37±0.04, 0.65±0.06, 0.71±0.06, 1.07±0.08 respectively, There was a significant difference that Group B compared with group A and group D, (p<0.05; n=6), There was not a significant difference that Group B compared with group C, (p>0.05; n=6).

**Figure 4:**
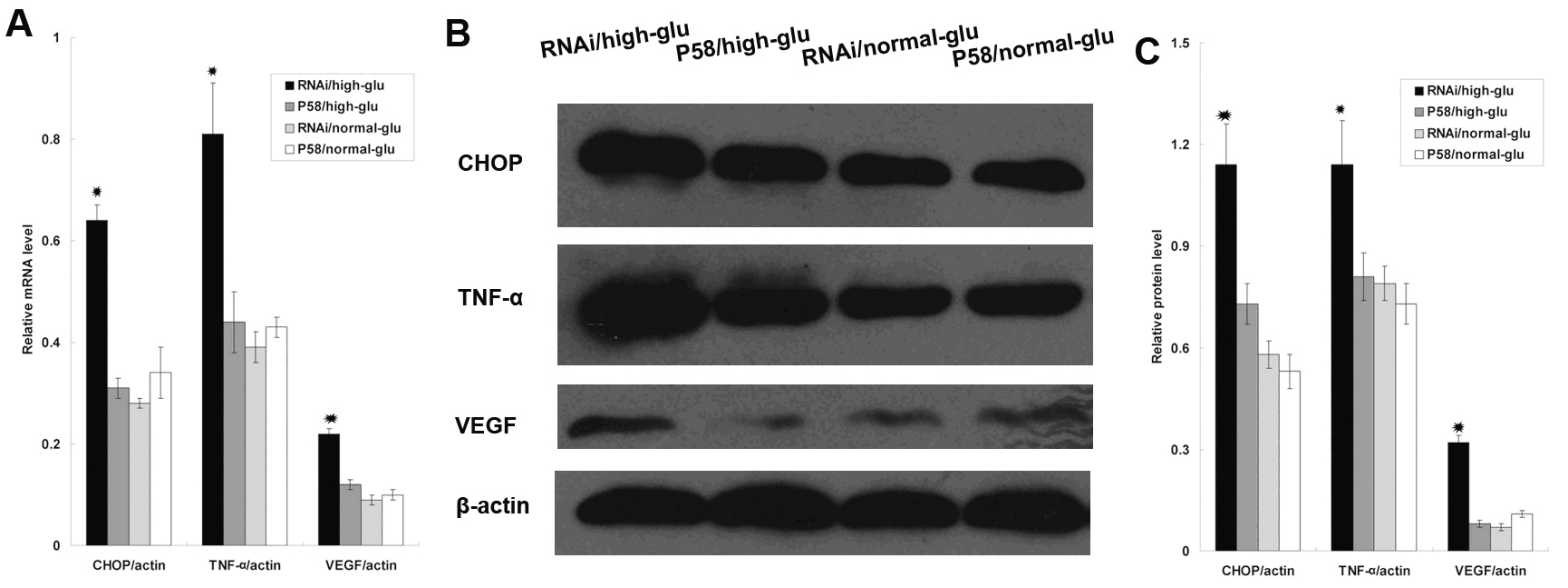
Overexpression of P58^IPK^ suppressed the expression of CHOP, TNF-α, and VEGF in cultured RRCECs. **A**: mRNA levels of CHOP, TNF-α, and VEGF were determined by real-time PCR, in GFP/dia group, GFP/no-dia group, P58^IPK^/dia group, and P58^IPK^/no-dia group. The expression level of CHOP in each groups were 0.64 ±0.03, 0.31±0.02, 0.28±0.01 and 0.34±0.05;respectively, (p<0.05, n=6), The expression level of TNF-α in each groups were 0.81±0.10, 0.44± 0.06, 0.39±0.03, and 0.43±0.02 respectively, (p<0.05, n=6), The expression level of VEGF in each groups were 0.22±0.01, 0.12±0.01, 0.09±0.01, and 0.10±0.01 respectively, (p<0.05, n=6),  **B**: A representative western blot result is shown in **B**. **C**: The amount of protein expression was quantified relative to the level of β-actin. Results are representative of six experiments. Results are expressed as mean±standard deviation; The protein level of CHOP in each groups were 1.14 ±0.12, 0.73±0.06, 0.58±0.04, and 0.53±0.05; respectively, (p<0.05, n=6), The protein level of TNF-α in each groups were 1.41±0.13, 0.81±0.07, 0.79±0.05, and 0.73±0.06 respectively, (p<0.05, n=6), The protein level of VEGF in each groups were 0.32±0.02, 0.08±0.01, 0.07±0.01 and 0.11±0.01 respectively, (p<0.05, n=6).

To further determine the effect of P58^IPK^ on the expression of CHOP, TNF-α, and VEGF expression, RRCECs were used. We first investigated the expression of P58^IPK^ in RRCECs that were transfected with pGIPZ-P58^IPK^ RNAi (group A), pGIPZ-GFP (control group, group B), irrelevant siRNA (group C), and rAAV2-P58^IPK^ (group D). No difference was detected between group C compared with group B. As shown in Figure 3A, the expression of P58^IPK^ in group D demonstrated enhanced P58^IPK^ mRNA expression, while P58^IPK^ RNAi decreased the P58^IPK^ mRNA expression significantly. Similar results were obtained in western blot analysis (Figure 3B,C). However, the expression of P58^IPK^ in group B did not increase, demonstrating that the endogenous P58^IPK^ was successfully inhibited by P58^IPK^ RNAi. Interestingly, P58^IPK^ overexpression downregulated the expression of CHOP, TNF-α, and VEGF in both mRNA and protein levels, while P58^IPK^ RNAi markedly upregulated the expression of CHOP, TNF-α, and VEGF in these cells compared to the control group (Figure 4), indicating that endogenous P58^IPK^ regulates the expression of CHOP, TNF-α, and VEGF in RRCECs.

## Discussion

ER stress activates various signaling pathways, which are termed the unfolded protein response. Three different classes of unfolded protein response branches, inositol-requiring protein-1, activating transcription factor-6, and protein kinase RNA-like ER kinase (PERK) [10], have been identified to mediate ER stress and trigger apoptosis in various diseases [11]. ER stress activates inflammatory responses [12,13] and leads to the onset of DR in diabetic patients [4,14]. P58^IPK^ is recognized as a 58-kDa inhibitor of the interferon-induced double-stranded RNA-activated protein kinase [5] and has been demonstrated to play an essential role in preventing ER stress [6,7]. The intracellular effect of P58^IPK^ on ER stress was achieved by inhibiting PERK activation [15], suggesting that P58^IPK^ is a key mediator of co-translocational ER protein degradation, and this process is likely to contribute to ER homeostasis in stressed cells. Mutant mouse strains with P58^IPK^ gene deletion displayed glucosuria, hyperglycemia, and hypoinsulinemia [16], suggesting that P58^IPK^ plays important roles in maintaining ER homeostasis and normal glucose of the body. In the present study, overexpression of P58^IPK^ significantly reduced blood vessel leakage in the retina of diabetic rats, indicating that elevated expression of P58^IPK^ may protect against diabetes-associated eye damage (Figure 1). Our data also demonstrated that overexpression of P58^IPK^ remarkably suppressed the mRNA and protein levels of CHOP, TNF-α, and VEGF compared with the control group (Figure 2B,C). In vitro studies using cultured RRCECs further confirmed that knocking down P58^IPK^ promoted both mRNA and protein expressions of CHOP, VEGF, and TNF-α, indicating a close relationship among P58^IPK^, CHOP, VEGF, and TNF-α.

A growing body of evidence has revealed that CHOP contributes to ER stress-induced retinal cell death [17,18]. Moreover, TNF-α-mediated apoptosis plays a pronominal role in the development of early DR [19]. Collectively, P58^IPK^ may relieve ER stress by maintaining ER functions, thus decreasing the levels of inflammatory factors and subsequently reducing the risks for DR development.

As the purpose of this paper is to verify the effect of P58^IPK^ protective retinal blood vessels in high glucose, we did not demonstrate that overexpressed P58^IPK^ affects the levels of PERK-signaling stress markers, such as PERK, phosphorylated eukaryotic initiation factor 2 (eIF2) and activating transcription factor 4 (ATF4). Future study will be focused on exploring the intracellular signaling pathway regulated by P58^IPK^. We found significant differences in the mRNA levels of CHOP and TNF between P58^IPK^/no-dia and GFP/no-dia groups; however, no significant differences were detected in the protein levels of CHOP and TNF between these two groups, suggesting that the effect of P58^IPK^ on CHOP and TNF may be limited to the transcriptional level, although the mechanisms need further study.

In summary, P58^IPK^ reduces the level of inflammatory factors, such as TNF-α. Our previous study confirmed that P58^IPK^ can reduce the expression of VEGFof retinal vascular endothelial cells [8] and therefore contributes to maintain homeostatic functions of ER. In addition, elevated expression of P58^IPK^ protects against ER stress-mediated apoptosis in vascular endothelial cells under high glucose conditions [8]. Thus, P58^IPK^ may act as a DR-resistant gene in diabetic patients.
